# Topological Dirac-vortex microcavity laser for robust on-chip optoelectronics

**DOI:** 10.1038/s41377-024-01398-1

**Published:** 2024-03-04

**Authors:** Yuanpeng Wu, Zetian Mi

**Affiliations:** Department of Electrical Engineering and Computer Science, University of Michigan, 1301 Beal Avenue, Ann Arbor, MI 48109 USA

**Keywords:** Lasers, LEDs and light sources, Nanophotonics and plasmonics

## Abstract

Dirac-vortex microcavity laser based on InAs/InGaAs quantum dots have been experimentally realized on silicon substrate. The topological laser features a large spectral range and high robustness against variations such as cavity size.

Semiconductor lasers that can be monolithically integrated on Si chips and have robust properties are highly desired for a broad range of applications^1,2^. Achieving such semiconductor lasers, however, has remained challenging. Dirac-vortex state^3^, a bond state in superconducting systems, have been shown to provide robust confinement of photons. The previously reported Dirac-vortex cavities are often limited to passive operations^4,5^. Over the past decade, significant improvements in the quality of III-V quantum dots (QDs) and the integration of QDs directly on Si substrate has been achieved^6–8^. A strategy of combining III-V QDs with the concept of topological laser is considered as a promising strategy for high-performance semiconductor lasers on the Si platform.

A recent publication by Ma et al. reported the design and fabrication of a Dirac-vortex topological laser based on InAs/InGaAs QD materials, which is monolithically integrated on Si substrate^9^. The Dirac-vortex photonic crystal laser cavity is designed to harness an auxiliary orbital degree of freedom in topological insulator. The Dirac-vortex topological laser is fabricated based on InAs/InGaAs quantum dots (QDs) epitaxially grown on an on-axis silicon (001) substrate. The photonic lattice was delineated in the active layer, which had a thickness of 362 nm, and was suspended by partially eliminating the 1-µm -thick Al_0.6_Ga_0.4_As sacrificial layer. This active layer effectively confines light in the vertical direction. In each unit cell, there are six triangular voids shown in Fig. 2b of this paper^9^, which can be categorized into two sets based on the C_3_ rotational symmetry. The separation from the centers of triangular voids to the unit cell’s center is ${\rm{d}}={a}_{0}/\sqrt{3\,}-{{\rm{\delta }}}_{t}$, where a non-zero δ_*t*_ disrupts the crystal’s T_P_ translational symmetry along the P vector. The polar coordinates (δ_0_, θ) were established using the expressions (δ_*t*_, δ_*i*_) = δ_0_ (α·sinθ, cosθ), where α = 0.65 (0.33) for δ_*t*_ > 0 (δ_*t*_ < 0). This configuration ensures that the bandgap at the Γ point exhibits reduced θ dependency. In this context, θ signifies an auxiliary orbital parameter useful for constructing the Majorana bound state (MBS)^10^. The effective bulk Hamiltonian is equivalent to the Jackiw–Rossi model. Using a three-dimensional finite-difference time-domain simulation, it is discovered that this apparatus sustains an MBS within the bulk bandgap, as illustrated in Fig. 2e. The computed modal pattern of this MBS with topological properties exhibits a mirror-symmetry relative to the dotted gray line, as depicted in Fig. 2f.

The micro-photoluminescence test was conducted employing a continuous-wave pump laser with a wavelength of 632.8 nm under room-temperature conditions. The relation between lasing peak intensity and the pump intensity I_pump_ indicates a critical pump intensity of I_th_ = 0.4 kW/cm^2^. Meanwhile, a reduction in spectral linewidth, affirming the operational state of lasing was observed. The normalized lasing spectra displayed in Fig. 3e reveals that the resonant wavelength of the topological Dirac-vortex lasers experiences a redshift as s_0_ decreases. This redshift effect can be utilized to tune the lasing wavelength across a large spectral range. The normalized experimental lasing spectra with distinct R_0_ values (the cavity size) affirms that the lasing wavelength stays at approximately 1340 nm. This arises because the Dirac-vortex cavity resonance is topologically fixed to the Dirac point, characterized by a null density of states, which contrasts with the nonzero density of states found in conventional structures, ensuring its independence from changes in R_0_.

In comparison to the Kekulé distortion approach^3^, this scheme is better suited for practical lasers, delivering linearly polarized emission rather than vector-beam output. Unlike conventional laser architectures such as vertical-cavity surface-emitting lasers (VCSELs) and Fabry–Pérot (FP) lasers^11,12^, the Dirac-vortex cavity lasers feature a fundamentally distinct scaling connection between their free spectral range (FSR) and modal volume V. In fact, conventional laser designs, including VCSELs and FP lasers, adhere to an FSR ~ V^-1^ relationship. In contrast, the Dirac-vortex cavity lasers follow an FSR ~ V^-1/2^ relationship, leading to a more substantial FSR for a given V. While some of the recent reports of solid-state lasers focuses on high-power^13^ and high-brightness^14^, Dirac-vortex lasers can achieve superior single-mode operation, and this advantage becomes increasingly evident as the modal volume V increases.

The measured far-field patterns match the simulated x-polarized, proving the lasing originates from the Dirac-vortex lasers with R_0_/a_0_ ratios of 0.01, 0.5, 1, and 2. It is crucial to consider the durability and consistency of the Dirac-vortex microcavity lasers. In essence, III–V quantum dots exhibit notable stability when properly shielded from direct exposure to the surrounding atmosphere^15,16^. In the experiments, however, quantum dots near the etched sidewalls lacked this protection, resulting in less stable devices due to photobleaching^17^. This concern can be resolved by applying a protective/passivation layer to mitigate the photobleaching impact.

The innovative accomplishment of Dirac-vortex microcavity lasers offers an opportunity to investigate topological incidents like non-Hermitian optics and quantum electrodynamics. The InAs/InGaAs based QD laser presents potential as an on-chip light source for forthcoming photonic integrated systems. Such exploration may result in notable progress within the realm of optoelectronics, establishing a route towards enhanced and resilient optical technologies. Moreover, demonstrating the capability of incorporating topological lasers seamlessly into a silicon substrate brings the community closer to the potential utilization of topological lasers in silicon-based optoelectronic platforms.
